# Supplementary material for: Stress-induced formation of cell wall-deficient cells in filamentous actinomycetes
Source: Nat Commun. 2018 Dec 4;9:5164. doi: 10.1038/s41467-018-07560-9 (PMC6279842; doi:10.1038/s41467-018-07560-9)
Supplement: Supplementary file 1 — Supplementary Information [file 41467_2018_7560_MOESM1_ESM.pdf]

Supplementary Information

Stress-induced formation of cell wall-deficient cells in filamentous actinomycetes

Ramijan *et al.*

Correspondence to: D.Claessen@biology.leidenuniv.nl

This PDF File includes:

Supplementary Figures 1-5

Supplementary Tables 1-8

Supplementary References

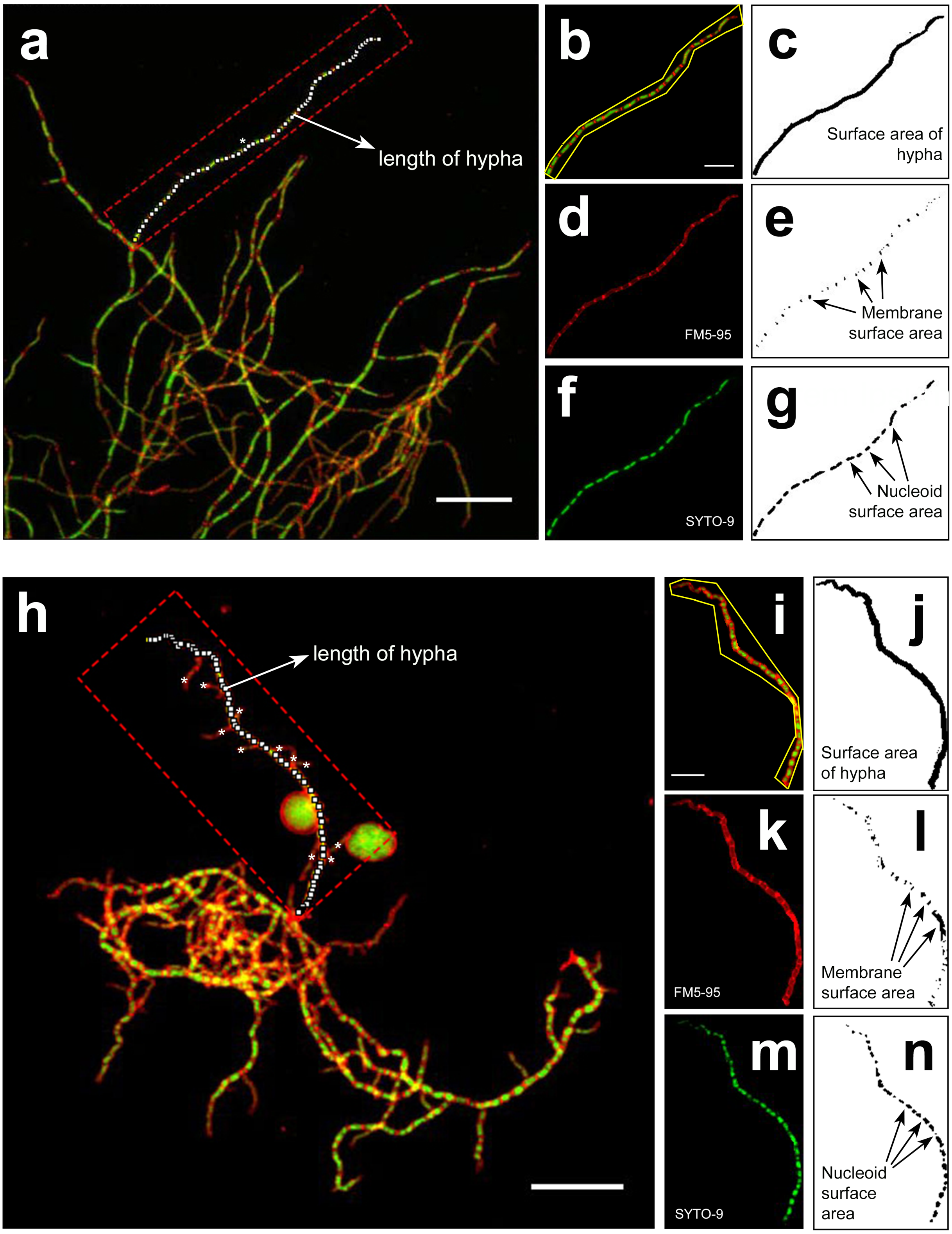

Supplementary Figure 1. Image analysis of *K. viridifaciens* hyphae.

Average Z-projection of a mycelial particle of *K. viridifaciens* grown in the absence (a) or presence (h) of 0.64 M sucrose. The red dashed rectangle shows the hypha that was selected for image analysis. Branches emerging from the leading hypha were counted and are indicated with asterisks. The length of the hypha was measured by drawing a segmented line and calculating its length using the FIJI software package. Selected hyphae were cropped from the pellet by drawing a polygon using FIJI (see yellow line in b, i). The merged pictures of the cropped hyphae were split into the separate channels to show the fluorescence derived from the FM5-95 membrane dye (d, k) and the fluorescence derived from the SYTO-9 DNA stain (f, m). The images corresponding to those of the red channel were duplicated to measure the hyphal surface area (c, j) and the surface area occupied by membranes (e, l). The green channel was used to measure the area occupied by individual nucleoids. Scale bars represent 20 µm (a, h) and 10 µm (b, i).

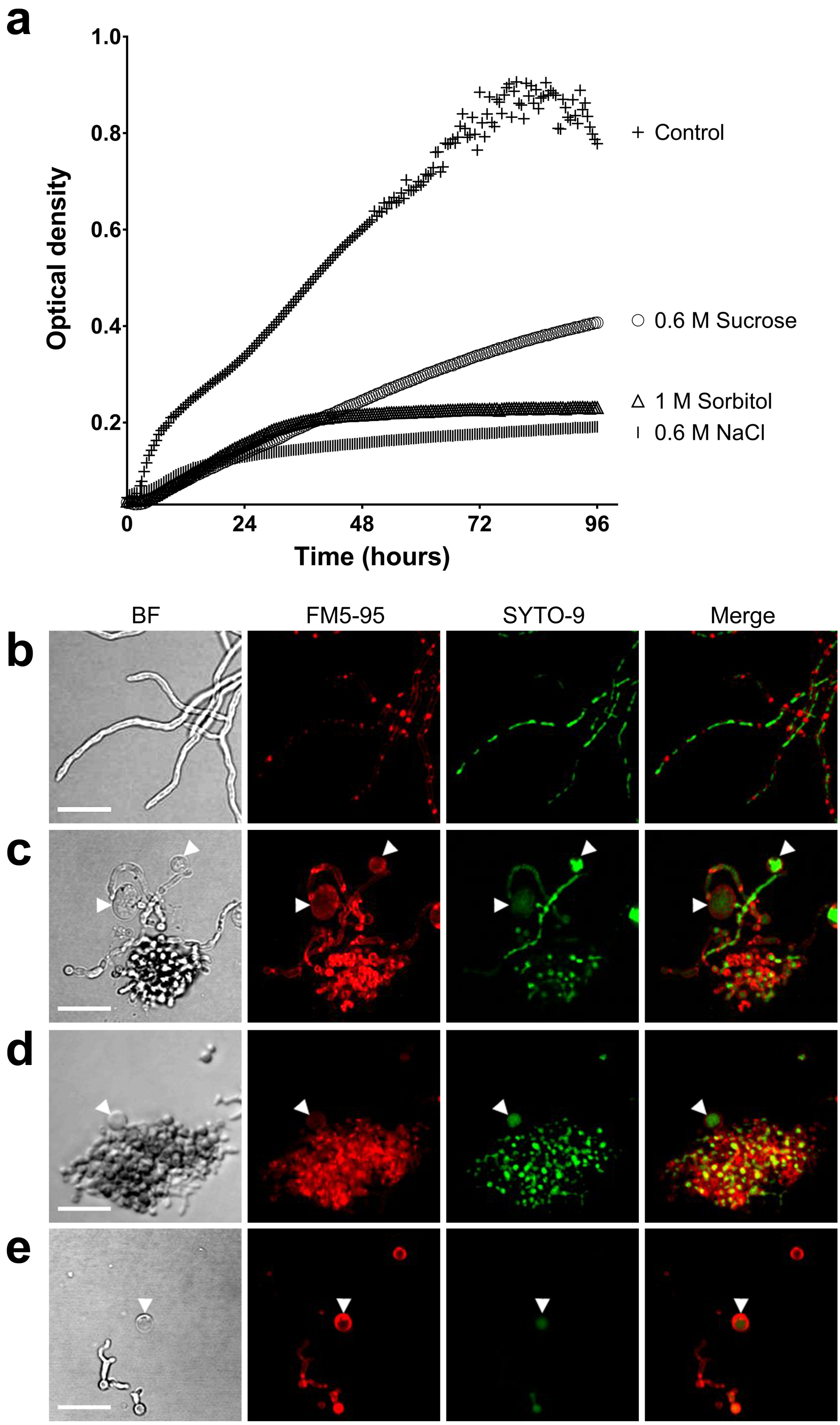

Supplementary Figure 2. High levels of osmolytes affect growth and lead to S-cell extrusion.
Growth curves of *K. viridifaciens* in LPB medium supplemented with different osmolytes (a). Values represent the average of five independent replicate cultures. Unlike in the absence of high levels of osmolytes (b), S-cells (arrowheads) were evident after 96 hours of growth in the presence of 0.6 M sucrose (c), 0.6 M NaCl (d) and 1 M sorbitol (e). Mycelium and cells were stained with FM5-95 and SYTO-9 to visualize membranes and DNA, respectively. Scale bars represent 10 µm.

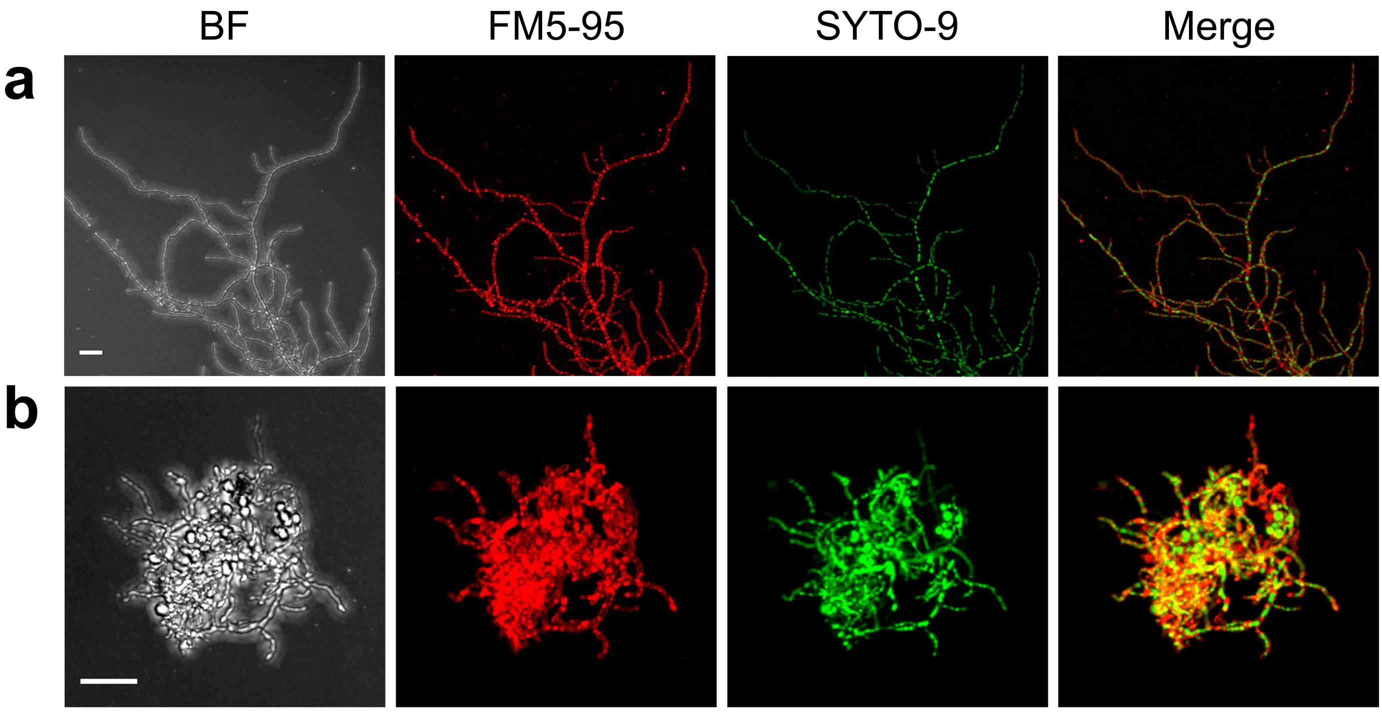

Supplementary Figure 3. High levels of salt affect pellet morphology in *K. viridifaciens*.
Mycelial morphology of *K. viridifaciens* grown in the absence (a) and presence (b) of 0.6 M NaCl after 48 hours. Mycelium was stained with FM5-95 and SYTO-9 to visualize membranes and DNA, respectively. Scale bars represent 10 µm.

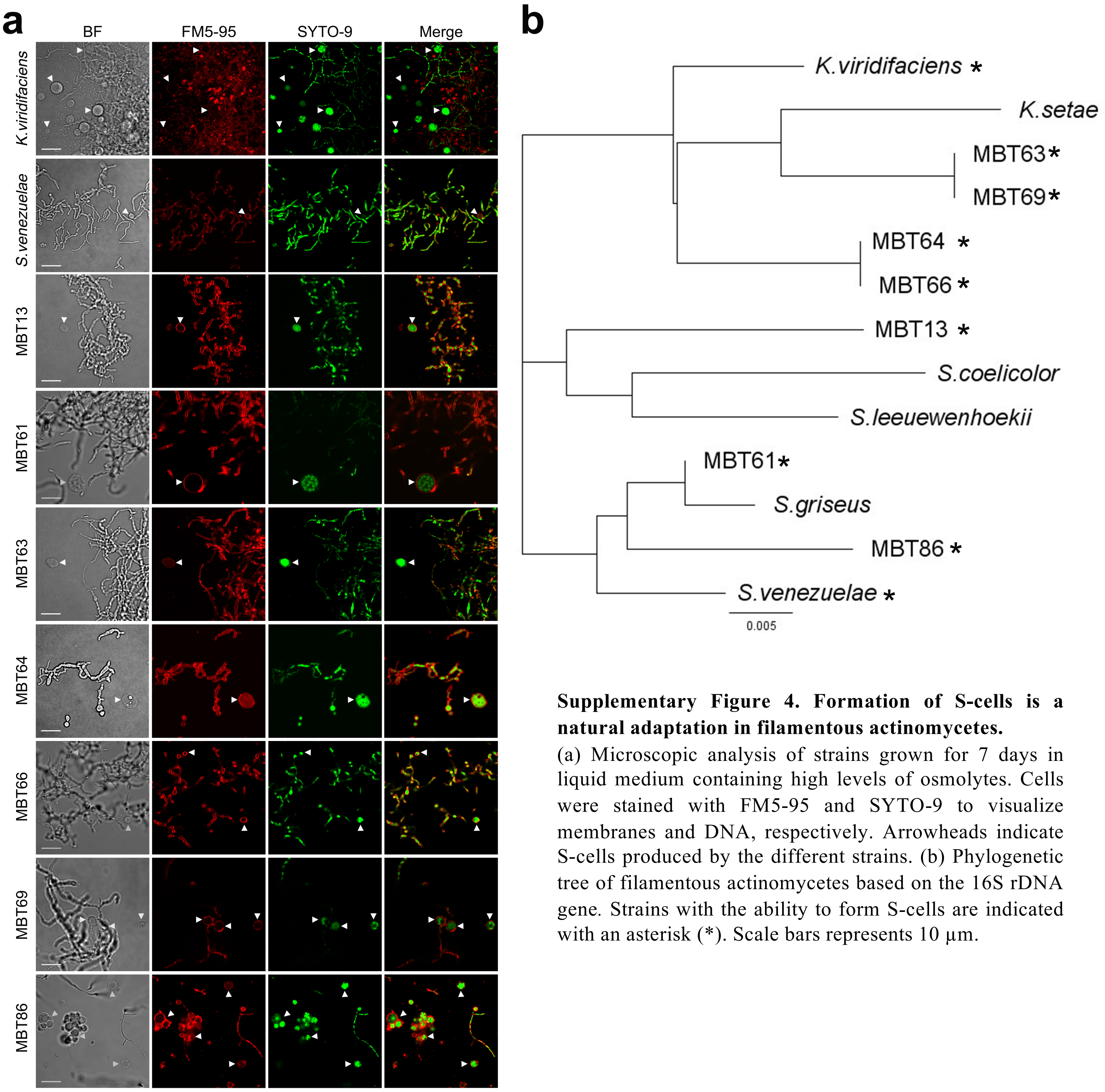

Supplementary Figure 4. Formation of S-cells is a natural adaptation in filamentous actinomycetes.

(a) Microscopic analysis of strains grown for 7 days in liquid medium containing high levels of osmolytes. Cells were stained with FM5-95 and SYTO-9 to visualize membranes and DNA, respectively. Arrowheads indicate S-cells produced by the different strains. (b) Phylogenetic tree of filamentous actinomycetes based on the 16S rDNA gene. Strains with the ability to form S-cells are indicated with an asterisk (*). Scale bars represents 10 µm.

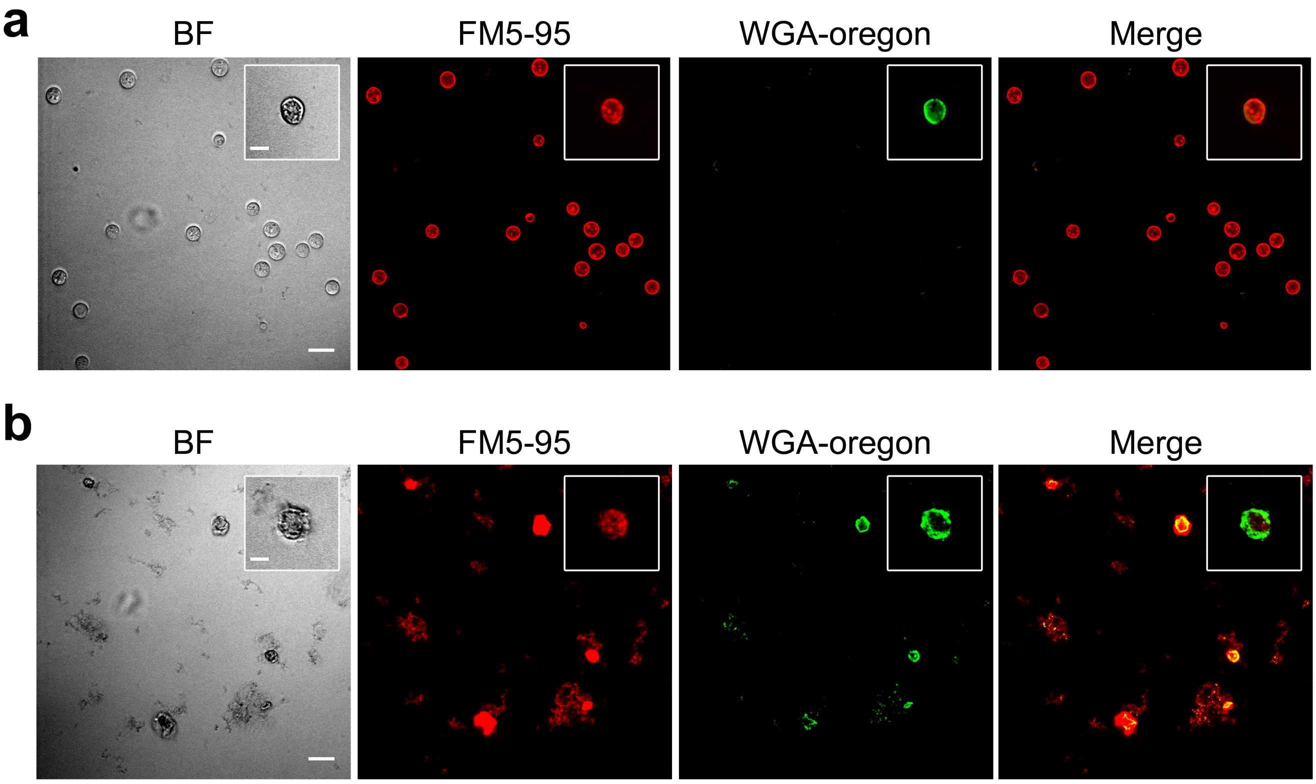

Supplementary Figure 5. Peptidoglycan surrounding S-cells confers resistance to water treatment.
(a) Filtered S-cells were stained with the membrane dye FM5-95 and WGA-Oregon to stain peptidoglycan. The inlay shows an S-cell possessing abundant cell wall material surrounding the cell surface. (b) Morphology of S-cells after exposing them to water. While many cells lyse, some S-cells remain intact, which invariably have abundant cell wall material associated with their cell surface (see inlay). Scale bars represents 10 µm, and 5 µm for the inlays.

Supplementary Table 1. Image analysis measurements on hyphae formed in the presence of low levels of osmolytes. SD represents the standard deviation. See Fig. S1 for details on the analysis.

Hypha	Length (µm)	Number of branches	Branching frequency [a]	Membrane fraction [b]	Average nucleoid area [c] (μm^2)
1	86,0180	1	0,01	0,10	2,53
2	90,377	3	0,03	0,09	2,01
3	72,3597	7	0,10	0,10	1,90
4	83,9838	0	0,00	0,10	3,02
5	79,0436	0	0,00	0,09	3,21
6	65,3853	1	0,02	0,17	4,18
7	80,7872	7	0,09	0,07	2,11
8	86,3086	2	0,02	0,10	3,05
9	76,4282	3	0,04	0,08	2,14
10	74,1033	2	0,03	0,12	1,71
Average	79,4795	2,6	0,03	0,10	2,59
SD	7,5857	2,5	0,03	0,03	0,77

[a] Branching frequency is the number of branches divided by the length of the hypha.
[b] Membrane fraction is the sum of the membrane surface area divided by the total area occupied by the hypha.
[c] Average nucleoid area represents the mean area of all the nucleoids present in the hypha.

Supplementary Table 2. Image analysis measurements on hyphae formed in the presence of high levels of osmolytes. SD represents the standard deviation. See Fig. S1 for details on the analysis.

Hypha	Length (μm)	Number of branches	Branching frequency [a]	Membrane fraction [b]	Average nucleoid area [c] (μm^2)
1	82,5308	11	0,13	0,22	1,35
2	90,9580	6	0,07	0,18	2,10
3	88,3428	8	0,09	0,16	1,82
4	60,4451	12	0,20	0,29	1,86
5	86,0180	9	0,10	0,24	2,08
6	76,7188	6	0,08	0,21	2,05
7	88,6334	6	0,07	0,22	2,34
8	53,4707	8	0,15	0,19	1,47
9	69,1631	7	0,10	0,20	2,04
10	70,3255	2	0,03	0,21	1,15
Average	76,6606	7,5	0,10	0,21	1,83
SD	12,9242	2,8	0,05	0,03	0,38

[a] Branching frequency is the number of branches divided by the length of the hypha.
[b] Membrane fraction is the sum of the membrane surface area divided by the total area occupied by the hypha.
[c] Average nucleoid area represents the mean area of all the nucleoids present in the hypha.

Supplementary Table 3. Calculated diameters (D) of S-cells released by different filamentous actinomycetes upon hyperosmotic stress. The diameters are indicated in µm.

Strain	D_{min}	D_{max}	D_{mean}	SD
K. viridifaciens	7,77	10,04	8,91	1,60
S. venezuelae	2,06	3,68	2,87	1,15
MBT13	2,62	4,49	3,56	1,32
MBT61	3,84	10,67	7,25	4,82
MBT63	2,52	5,21	3,86	1,91
MBT64	2,12	4,99	3,55	2,03
MBT66	2,56	6,78	4,67	2,99
MBT69	3,87	6,27	5,07	1,70
MBT89	2,01	6,05	4,03	2,86

Supplementary Table 4. Mutations in the hyperosmotic stress-induced L-form strain M1

Variation	Position	Type	Reference	Allele	Locus	Protein	Effect in protein
1	4456932	SNV	C	G	BOQ63_RS28320	Acetyltransferase	Leu99Val
2	4876534	SNV	T	C	BOQ63_RS30295	Valine-tRNA ligase	Val319Ala
3	3219590	SNV	C	G	NCR		
4	3133612	SNV	G	A	BOQ63_RS21920	Metal ABC transporter ATPase	Asp504Asn

SNV: Single Nucleotide Variation

Supplementary Table 5. Mutations in the hyperosmotic stress-induced L-form strain M2

Variation	Position	Type	Reference	Allele	Locus	Protein	Effect in protein
1	2164717	SNV	A	G	NCR		
2	5054842	SNV	T	G	BOQ63_RS31145	XRE family transcriptional regulator	Glu332Ala
3	6460621-6460623	Deletion	CCA	-	BOQ63_RS37840	Histidine kinase	Thr606del
4	3 133753^ 3133754	Insertion	-	C	BOQ63_RS21920	Metal ABC transporter ATPase	Arg553fs

SNV: Single Nucleotide Variation, del: deletion, fs: frame shift

Supplementary Table 6. Mutations in the penicillin-induced L-form

Variation	Position	Type	Reference	Allele	Locus	Protein	Effect in protein
1	546832	SNV	C	A	NCR		
2	3549271	SNV	G	A	BOQ63_RS23890	Lysylphosphatidylglycerol synthetase-like protein	Thr203Ile
3	3297354	SNV	C	A	BOQ63_RS22750	Undecaprenyl-diphosphate phosophatase	Leu58Met

SNV: Single Nucleotide Variation

Supplementary Table 7. Strains used in this study

Strains	Genotype	Reference
***Streptomyces/Kitasatospora* strains**		
Streptomyces coelicolor A3(2) M145	Wild-type	Lab collection
Streptomyces lividans 1326	Wild-type	Lab collection
Streptomyces griseus	Wild-type	Lab collection
Streptomyces venezuelae DIVERSA	Wild-type	Lab collection
Kitasatospora viridifaciens DSM40239	Wild-type	DSMZ[1]
K. viridifaciens Δ*ssgB*	DSM40239 *ssgB::apra*	This work
Streptomyces sp. MBT13	Wild-type	Lab collection[2]
Streptomyces sp. MBT61	Wild-type	Lab collection[2]
Kitasatospora sp. MBT63	Wild-type	Lab collection[3]
Kitasatospora sp. MBT64	Wild-type	Lab collection[2]
Kitasatospora sp. MBT66	Wild-type	Lab collection[3]
Kitasatospora sp. MBT69	Wild-type	Lab collection[2]
Streptomyces sp. MBT86	Wild-type	Lab collection[2]
***K. viridifaciens* cell wall-deficient strains**		
Penicillin-induced L-form	Mutant	This work
Hyperosmotic stress-induced L-form M1	Mutant	This work
Hyperosmotic stress-induced L-form M2	Mutant	This work

Supplementary Table 8. Primers used in this study

**Primer**	**Sequence (5' – 3')**
Consensus_*ssgB*-Fw	ATGAACACCACGGTCAGCTG
Consensus_s*sgB*-Rv	GCTCTCGGCCAGGATGTG
P1-*ssgB*-FW	GACGAATTCAGGCGTCAGAAACGGGTATC
P2-*ssgB*-RV	GAAGTTATCCATCACCTCTAGAGCTGACCGTGGTGTTCATAAGC
P3-*ssgB*-FW	GAAGTTATCGCGCATCTCTAGACTGAGCTCTCCGGAAGGAGAA
P4-*ssgB*-RV	GACAAGCTTTCTACCTGACCGGGCTGTT
qPCR_*infB*-Fw	GTCACGTCGACCACGGTAAG
qPCR_*infB*-Rv	CACCGATGTGCTGGGTGATG
qPCR_*atpD*-Fw	TTCGGACAGCTCGTCCATAC
qPCR_*atpD*-Rv	ACATCGCGCAGAACCACTAC
qPCR_*parA*-Fw	CGGTCGTCACCCAGTACAAG
qPCR-*parA*-Rv	TAACCGAGTTCGAGGGACAG
qPCR-*Orf1*-Fw	GAGGGAGCCAATCCCGTATC
qPCR-*Orf1*-Rv	GGCTGTTGGACAGGACCATC
qPCR-*allC*-Fw	CGGCGATAGCGGAGACTAAG
qPCR-*allC*-Rv	CCACTGGTGGGACCAGAAAG
qPCR-*tetR*-Fw	TGCTCGACCAGCTGTTGAAG
qPCR-*tetR*-Rv	TGGCGAGCATGAAGTCGTAG
BOQ63_RS28320-Fw	CTAGGTCGAAGGACCGATGG
BOQ63_RS28320-Rv	CGGACGTGACGCTCTACAAC
Seq_RS28320-Rv	GAAATCGGCCAGCGGGTAAG
Seq_RS30295-Fw	CTTCAAGCGCCTGTTCGACG
Seq_RS30295-Rv	TGTCGACCCAGTCGAAGTAG
Seq_NCR-M1-Fw	CGTTGCGGATGTGGTTCTTG
Seq_NCR-M1-Rv	GTTCGCTGGCCGAGATGTTC
Seq_RS21920-Fw	TGATCGAGGCGATGCCCTTC
Seq_RS21920-Rv	CGTTCGATGTTGCCGATCAC
Seq_NCR-M2-Fw	AGAGCAGCATGCCGAGCTTG
Seq_NCR-M2-Rv	CTTCCTTGGTCGGGAAGTAG
Seq_RS31145-Fw	GTGGTGAATCCGTGCCACAG
Seq_RS31145-Rv	TGGAACGCCTACTCCATGGG
Seq_RS37840-Fw	GATCTCCACGCCGTTGAAAG
Seq_RS37840-Rv	GAGTTCGGTGGTTTCGAAGG
Seq_NCR-L-form-Fw	GTGGCTCATTCAGGACTCTC
Seq_NCR-L-form-Rv	CGCCGCTTCATCTCTGATAC
Seq_RS23890-Fw	GAGAAGATCACCGCCTTGTC
Seq_RS23890-Rv	ACAGGCACCCGCTCAACTAC
Seq_RS22750-Fw	CCGGTGACACCCGGAAATAC
Seq_RS22750-Rv	CCGGGATGGTGGAGATGATG

Supplementary References

1. Ramijan, K., van Wezel, G. P. & Claessen, D. Genome sequence of the filamentous actinomycete *Kitasatospora viridifaciens*. *Genome Announc.* **5**, e01560-16 (2017).
2. Zhu, H. *et al.* Eliciting antibiotics active against the ESKAPE pathogens in a collection of actinomycetes isolated from mountain soils. *Microbiology* **160**, 1714-1725 (2014).
3. Girard, G. *et al.* Analysis of novel kitasatosporae reveals significant evolutionary changes in conserved developmental genes between *Kitasatospora* and *Streptomyces*. *Antonie Van Leeuwenhoek* **106**, 365-380 (2014).
