# Supplementary material for: Stress-induced formation of cell wall-deficient cells in filamentous actinomycetes
Source: Nat Commun. 2018 Dec 4;9:5164. doi: 10.1038/s41467-018-07560-9 (PMC6279842; doi:10.1038/s41467-018-07560-9)
Supplement: Supplementary file 3 — Description of Additional Supplementary Files [file 41467_2018_7560_MOESM3_ESM.pdf]

Description of Additional Supplementary Files

File Name: Supplementary Movie 1
Description: Apical extrusion of S-cells in K. viridifaciens. S-cells are extruded from the hyphal tip after 425 min, coinciding with a transient arrest in tip growth. After extrusion of S-cells, a new tip is formed in the apical region of the hyphae after 540 min, while subapically new branches become visible after 620 min. The times are indicated in min. The scale bar represents 10 µm.

File Name: Supplementary Movie 2
Description: Extrusion of S-cells from branches in K. viridifaciens. S-cells are extruded from the tips of branches that are formed subapically. The times are indicated in min. The scale bar represents 10 µm.

File Name: Supplementary Movie 3
Description: S-cells of K. viridifaciens contain DNA and inner membrane assemblies. Z-stack projections of S-cells isolated after 48 hours, which were stained with Hoechst (blue) and FM5-95 (red) to visualize DNA and membranes, respectively. The scale bar represents 10 µm.

File Name: Supplementary Movie 4
Description: Switching of ΔssgB S-cells to the mycelial mode-of-growth. Switching of an S-cell of the ΔssgB mutant on solid R5 medium yields a mycelial colony. The times are indicated in min. The scale bar indicates 20 µm.

File Name: Supplementary Movie 5
Description: Example of vesiculation during proliferation of strain M2. Time-lapse microscopy showing proliferation of strain M2 on media containing high levels of sucrose. Please note that vesiculation is evident in some cells. The times are indicated in min. The scale bar indicates 5 µm.

File Name: Supplementary Movie 6
Description: Example of blebbing during proliferation of strain M2. Time-lapse microscopy showing proliferation of strain M2 on media containing high levels of sucrose. Please note that blebbing is evident in some cells. The times are indicated in min. The scale bar indicates 5 µm.

File Name: Supplementary Movie 7
Description: Example of membrane tubulation during proliferation of strain M2. Time-lapse microscopy showing membrane tubulation in strain M2 during proliferation on media containing high levels of sucrose. The times are indicated in min. The scale bar indicates 5 µm.
